# Supplementary material for: The Effects of Ozone on Atlantic Salmon Post-Smolt in Brackish Water—Establishing Welfare Indicators and Thresholds
Source: Int J Mol Sci. 2020 Jul 20;21(14):5109. doi: 10.3390/ijms21145109 (PMC7404298; doi:10.3390/ijms21145109)
Supplement: Supplementary file 1 [file ijms-21-05109-s001.pdf]

**Supplementary data**

## Epidermis

| Epidermis | Score | Description |
|---|---|---|
| General appearance | 0 | Even epidermis all over |
| | 1 | Uneven epidermis |
| | 2 | Parts of the epidermis is missing |
| | 3 | Most of the epidermis is missing |
| | | |
| **Epidermis** | **Score** | **Description** |
| Surface | 0 | Smooth suface |
| | 1 | Signs of rough sells at the surfaace |
| | 2 | Clear signs of rough cells, <50% of the surface affected |
| | 3 | All cells lining the oter part of the epidermis appears rough |

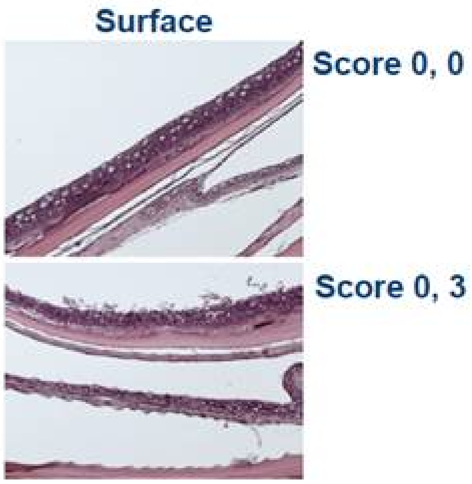


**S1:** Skin scoring table for general and surface appearance of the Epidermis after Sveen, 2018.

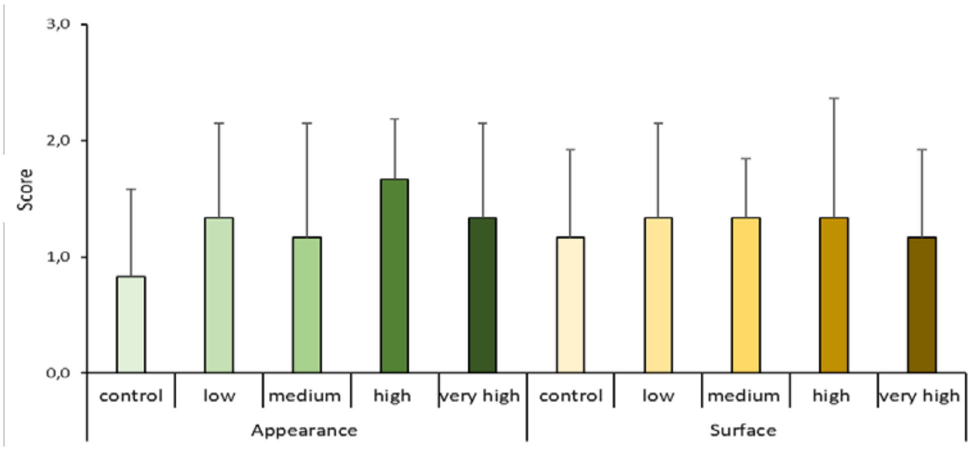


**S2:** Skin morphology scores of salmon exposed to different ozone treatments. Skin health status was assessed based on the general appearance and the surface appearance using a 3-point scale scoring system (0 indicates healthy skin with even and smooth cutaneous surface and epithelial structures are intact while 3 denotes poor skin health with epidermis missing and rough surface). Values are presented as mean ± SD of 6 individual fish per treatment group. No statistical difference between groups.
